# Supplementary material for: Is radiographic lumbar spinal stenosis associated with the quality of life?: The Wakayama Spine Study
Source: PLoS One. 2022 Feb 17;17(2):e0263930. doi: 10.1371/journal.pone.0263930 (PMC8853503; doi:10.1371/journal.pone.0263930)
Supplement: Attachment — Submitted filename: report.docx [file pone.0263930.s002.docx]

**Report on**

A large-scale, population-based cohort study in Japan to determine the effect of lumbar spinal stenosis seen on MRI on the quality of life: The Wakayama Spine Study

or

Effect of lumbar spinal stenosis seen on MRI on the quality of life

**General Comments:**

Lumbar spinal stenosis is a narrowing of the spinal canal in the lower part of the patient's back. The manuscript aimed to aimed to determine the association between radiographic lumbar spinal stenosis (LSS) and the quality of life (QOL) in the general Japanese population.

907 patients were clustered into 4 groups no, mild, moderate, and severe stenosis. The study was prospective which is a very good advantage.

Exclusion Criteria:

1. Patients younger than 40 years.
2. Patients who had undergone previously to lumber spine surgery.

In this general population study, severe radiographic LSS was associated with LBP, but did not affect ODI.

The topic is interesting and is of relevance to the readers of the journal.

**Introduction**

The introduction was well organized and fully explained.

**Methodology**

1. Description of the experimental procedures was concise and contained detailed descriptions of well-established procedures.
2. The number of patients was sufficient.
3. Inclusion and Exclusion criteria seem logical.

**Results and Discussion**

1. The results were represented by nice and informative radiographic images showing LSS.
2. The study findings were adequately discussed.

**Decision**

The study is interesting. Therefore, I recommend it for publication as it is.

Amr A. Abd-Elghany

Associate Professor,

Radiology and Medical Imaging Department,

College of Applied Medical Sciences
