# Supplementary material for: Is radiographic lumbar spinal stenosis associated with the quality of life?: The Wakayama Spine Study
Source: PLoS One. 2022 Feb 17;17(2):e0263930. doi: 10.1371/journal.pone.0263930 (PMC8853503; doi:10.1371/journal.pone.0263930)
Supplement: Attachment — Submitted filename: response to reviewers.docx [file pone.0263930.s003.docx]

January 10, 2022

Emily Chenette

Editor-in-Chief

PLOS ONE

Re: Is radiographic lumbar spinal stenosis associated with the quality of life?: The Wakayama Spine Study

Dear Editor:

Thank you for giving us the opportunity to revise the above-named manuscript in light of the comments made by the reviewers. Please find attached a clean revised manuscript, a marked-up revised manuscript, and a response to review document.

We sincerely hope that you will find that the revised manuscript merits publication in the *PLOS ONE* in due course.

Sincerely,

Yuyu Ishimoto, MD, PhD,

Wakayama Medical University,

811–1 Kimidera, Wakayama City,

Wakayama 641–8509, Japan.

E-mail:yuyu.ishimoto@hotmail.co.jp

Fax:1 073-447-2300

Reviewer #1: Report on

>Thank you very much for your comment.

Reviewer #2: 

2. The title is wordy, so it could be more concise.
>Thank you for your advice. I have changed the title as you said.

“Is radiographic lumbar spinal stenosis associated with the quality of life?: The Wakayama Spine Study”

3. In abstract, the font size is not consistent. And in methods, logistic regression analysis was not mentioned but in results, it was used in fact.

>I corrected the font size in abstract. I also added the sentence about logistic regression analysis into the methods.

4. In the Introduction, the words "radiographic LSS and the quality of life (QOL) has not been investigated in the Japanese general population.. Therefore, we aimed to assess the effect of radiographic LSS on the QOL using mobile magnetic resonance imaging (MRI) and the Oswestry Disability Index (ODI) in them" is not scientific, and more importantly, in the discussion part (paragraph 3) described that "there are vary reports on the relationship between radiographic LSS and QOL.."

>Thank you for your comment. In fact, there have been several studies on the relationship between radiographic LSS and the QOL, though, most their subjects were patients who were outpatients or underwent surgery. We could not find the study on the association of LSS with QOL using general people.　I deleted above sentence and added another sentence in the introduction.

5. How many cases were excluded and the reason?
> Thank you for your comment. I added the some sentences in the methods for clarity. Firstly, 1,063 volunteers were recruited for MRI from the second visit ROAD study. Fifty-two of these declined to attend the examination, and the remaining 1,011 were registered in the Wakayama Spine Study. Participants who had sensitive implanted devices (such as a pacemaker) or other disqualifiers were excluded. In A total of 977 participants underwent a lumbar spine MRI in a mobile MRI unit. Ten participants who underwent a previous lumbar surgery for LSS and 29 participants aged <40 years were excluded from the study because LSS is a degenerative disease.

6. As this is a large-scale clinical study, some useful clinical relevance in Conclusion would be more attractive.

>Thank you for your comment. I added the below sentences in Conclusion.

However, it is also true that some of the mild cases may become more severe and lead to surgery. Further longitudinal surveys of The Wakayama Spine Study will help to further clarify the aggravating factor for LBP and QOL.

Reviewer #3: 
Abstract:
Page 6 Line 91: The prevalence of severe, moderate, and non-mild/non-radiographic ..........This sentence incomplete

>Thank you very much. I completed the sentence.

Line 100: LBP2: Abbreviation should be written in complete form when mentioned for the first time in the manuscript

>Thank you very much. I corrected as you mentioned.

Materials and Methods:
Page 11 Line 182-187: Number of patient is incorrect (It should be938 patient). You should explain this.

> Your point is right. I corrected the number and added some sentences for clarity.

Fig. 5. The figure should be corrected.

>Thank you for your advice. I corrected the title of Fig.5 and the names for the vertical and horizontal axes to make it easier to understand. In addition, I changed the color of bars. (The more severe stenosis, the darker the color)

I also added the last sentence about Q8 associated sexual function.
